# Disentangling the Progression of Non-alcoholic Fatty Liver Disease in the Human Gut Microbiota

**DOI:** 10.3389/fmicb.2021.728823

**Published:** 2021-10-13

**Authors:** Tianjiao Wang, Xue-Kun Guo, Huji Xu

**Affiliations:** ^1^School of Medicine, Tsinghua University, Beijing, China; ^2^Department of Rheumatology and Immunology, Shanghai Changzheng Hospital, The Second Military Medical University, Shanghai, China; ^3^Peking-Tsinghua Center for Life Sciences, Tsinghua University, Beijing, China

**Keywords:** liver cirrhosis, liver fibrosis, meta-analysis, microbiome, NAFLD

## Abstract

Gut microbiome dysbiosis has been known to be associated with all stages of non-alcoholic fatty liver disease (NAFLD), but questions remain about microbial profiles in progression and homogeneity across NAFLD stages. We performed a meta-analysis of three publicly shotgun datasets and built predictive models to determine diagnostic capacity. Here, we found consistently microbiome shifts across NAFLD stages, of which co-occurrence patterns and core sets of new biomarkers significantly correlated with NAFLD progression were identified. Machine learning models that are able to distinguish patients with any NAFLD stage from healthy controls remained predictive when applied to patients with other NAFLD stages, suggesting the homogeneity across stages once again. Focusing on species and metabolic pathways specifically associated with progressive stages, we found that increased toxic metabolites and decreased protection of butyrate and choline contributed to advanced NAFLD. We further built models discriminating one stage from the others with an average of 0.86 of area under the curve. In conclusion, this meta-analysis firmly establishes generalizable microbiome dysbiosis and predictive taxonomic and functional signatures as a basis for future diagnostics across NAFLD stages.

## Introduction

Non-alcoholic fatty liver disease (NAFLD) is defined as the pathological accumulation of lipid droplets in >5% of hepatocytes (Sberna et al., 2018), developing from simple non-alcoholic fatty liver (NAFL), progressing toward non-alcoholic steatohepatitis (NASH), which can also present with liver fibrosis, the main prognostic lesion for disease progression, and ultimately leading to cirrhosis or hepatocellular carcinoma (Fingas et al., 2016). It is rapidly becoming the most prevalent liver disease and also the most increasing cause of hepatocellular cancer and liver transplantation in Western countries. Liver biopsy is the diagnostic gold standard to assess the disease severity, yet it is an invasive, traumatic, inconvenient tool, making it unfeasible for disease screening, diagnosis, or examining progression in routine care. Early identification of the presence of the advanced stage of NAFLD using non-invasive form is a major unmet need in the field.

Evidence is accumulating that the gut microbiome is involved in the etiology of NAFLD, while few studies have focused specifically on microbiota signatures in NAFLD at species level (Qin et al., 2014; Loomba et al., 2017; Hoyles et al., 2018) and even fewer have examined microbial composition as a function of NAFLD progression (Caussy et al., 2019). Qin et al. (2014) characterized the gut microbiome in liver cirrhosis, but this study involved diverse etiologies of cirrhosis (such as alcoholic liver disease, hepatitis B, and hepatitis C) and did not provide gut microbiome signatures that are specific to NAFLD-related cirrhosis. There are also studies comparing different stages of liver steatosis (Hoyles et al., 2018) or fibrosis severity (Boursier et al., 2016; Loomba et al., 2017; Shen et al., 2017), yet these stages are not exactly the same as the stage of disease progression. Caussy et al. (2019) explored patients with non-NAFLD, NAFLD without advanced fibrosis, or NAFLD cirrhosis, while most of the identified promising diagnostic signatures were unknown genera. Importantly, researches have mainly compared the differences between NAFLD patients and healthy controls or between mild stage and advanced stage of fibrosis, the generalizable microbiome dysbiosis across stages has been ignored. Thus, the alteration of the gut microbiome in NAFLD progression and the generality of microbiome dysbiosis across stages urgently need to be demonstrated.

Here, we collected currently available NAFLD shotgun metagenomic datasets (Boursier et al., 2016; Loomba et al., 2017; Shen et al., 2017), re-classified 107 patients with NAFL, NASH, fibrosis, or cirrhosis and 120 healthy controls according to clinical diagnosis, performed an integrated analysis combining all datasets, and assessed prediction accuracies of the gut microbiome for the detection of the different stages in NAFLD progression. Our study aims to find a panel of gut microbiome-derived biomarkers generally associated with NAFLD across stages or linking NAFLD progression, which help to develop a non-invasive diagnosis of NAFLD.

## Results

### Gut Microbiome Changes in Non-alcoholic Fatty Liver Disease Progression

**FIGURE 1:**
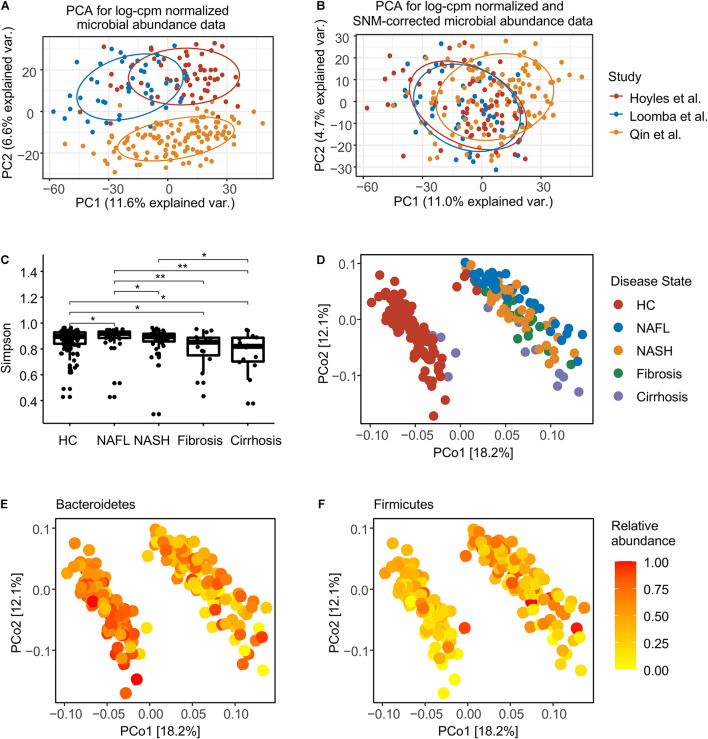
Normalization and overview of the NAFLD microbiome taxonomic profiles at species level. **(A)** Principal components analysis (PCA) of log-cpm normalized data, with NAFLD microbiome samples colored by studies. **(B)** PCA of log-cpm-SNM data. **(C)** Alpha diversity for each disease stage. The differences were calculated by two-sided Wilcoxon test. *p < 0.05; **p < 0.01. **(D)** Principal coordinates analysis (PCoA) based on species-level Bray–Curtis dissimilarity colored by disease stage. **(E)** PCoA colored by the normalized abundance of phylum Bacteroidetes. **(F)** PCoA colored by the normalized abundance of phylum Firmicutes. HC, healthy controls; NAFL, non-alcoholic fatty liver; NASH, non-alcoholic steatohepatitis.

After filtering samples with incomplete diagnostic information, we considered 39 patients with NAFL, 39 patients with NASH, 15 patients with fibrosis, 14 patients with cirrhosis, and 120 healthy controls (Supplementary Table 1). In total, assembled sequences for 493 species (Supplementary Table 2), 1,718,123 gene families (Supplementary Table 3), and 432 metabolic pathways (Supplementary Table 4) were identified in the 227 samples. The combined metagenomic data showed substantial batch effects (Figure 1A), so we converted discrete taxonomical counts into log-counts per million (log-cpm) per sample, and performed supervised normalization (SNM) (Qin et al., 2012) to reduced batch effects (Figure 1B). We then contrasted the effect of disease-associated heterogeneity on microbiome composition with potential confounders [patient age, body mass index (BMI), and sex], and this analysis revealed BMI to have an impact on species composition as predominant as disease (Supplementary Figure 1A), since patients with NAFLD often present with obesity. To address this issue, for the present study, a methodology was chosen that explicitly models BMI as a confounder in all applicable tests (Hothorn et al., 2006). Consistent with prior studies (Caussy et al., 2019; Ponziani et al., 2019), samples from individuals with more serious NAFLD stage had significantly lower Simpson alpha diversity (Figure 1C). The stage of health or disease contributed to the first axis of species-based principal coordinates (Figure 1D), and the most variation is driven by a trade-off between phylum Bacteroidetes and Firmicutes (Figures 1E,F and Supplementary Figure 1B).

### Univariate Analysis of Species Associated With Non-alcoholic Fatty Liver Disease

**FIGURE 2:**
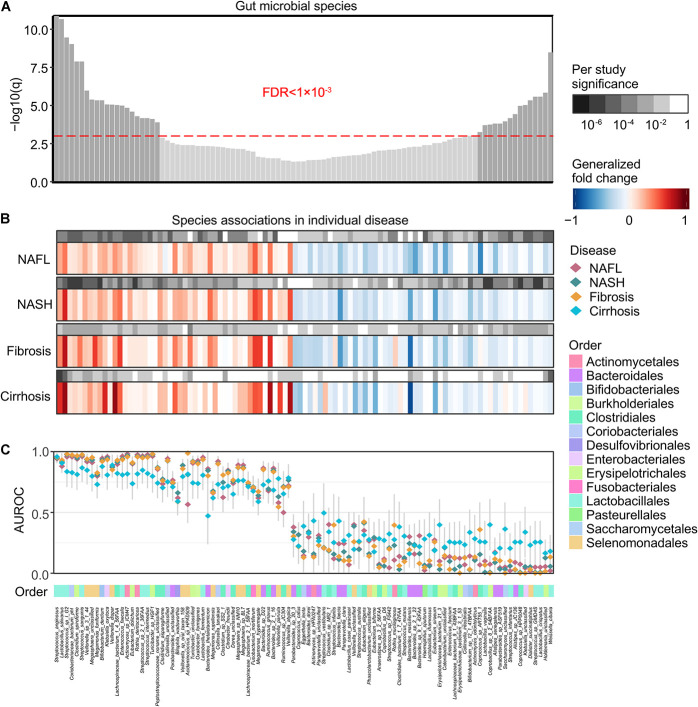
Meta-analysis identifies a core set of gut microbes strongly associated with NAFLD. **(A)** Meta-analysis significance of gut microbial species derived from blocked Wilcoxon tests is given by the bar height (FDR = 0.05). Bars of a core of highly significant species (meta-analysis FDR = 1 × 10^– 3^) were colored as dark gray. **(B)** Species-level significance, as calculated with a blocked two-sided Wilcoxon test (FDR *P* value), and the generalized fold change within individual stages are displayed as heatmaps in gray and in color, respectively. Species are ordered by meta-analysis significance and direction of change. NAFL, non-alcoholic fatty liver; NASH, non-alcoholic steatohepatitis. **(C)** Association strength is quantified by the AUROC across individual stages (color-coded diamonds), and the 95% confidence intervals are indicated by the gray lines. Order-level taxonomic information is color-coded above the species names.

At a meta-analysis FDR of 0.05, we identified 99 species, out of 261 species consistently detected across studies, to be associated with general NAFLD microbiome dysbiosis, of which 47 microbial species were identified to be significantly enriched in patients and 52 microbial species were identified to be significantly depleted (Figure 2A). The gut microbial community had consistent alteration patterns across different disease stages (Figure 2B and Supplementary Figure 2), and the majority of significant species were compatible identified as significant in individual disease stages (Figure 2B). Our results are easily reconciled with a model in which increased pathogenic microbes and a lack of protective microbes contribute to NAFLD development (Figure 2C). These species showed relatively low accuracy for cirrhosis, and this may be partially due to the low sample size.

**FIGURE 3:**
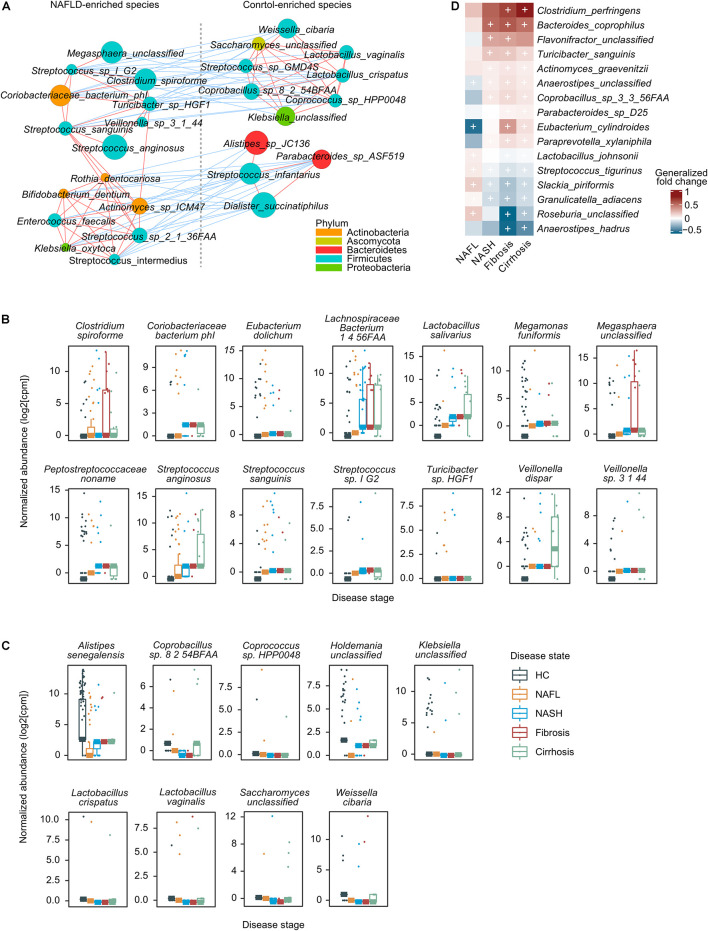
A co-occurrence network and the correlation between gut microbes and NAFLD. **(A)** A co-occurrence network for the core set of highly significant species. The size of the nodes indicates normalized abundance. The color of the nodes indicates their taxonomic assignment. Connecting lines represent Spearman correlation coefficient values above 0.8 (red) or below −0.8 (blue). **(B)** Boxplots showing that species was significantly correlated with NAFLD stages using partial Spearman’s rank-based correlation (pSRC), adjusted for BMI (two-sided, FDR < 0.05). **(C)** Boxplots showing that species was significantly anti-correlated with NAFLD stages (pSRC, two-sided, FDR < 0.05). **(D)** Heatmap showing the species that significantly and consistently changed in the last three stages (blocked two-sided Wilcoxon tests, + indicates statistical significance FDR < 0.05). Generalized fold change is colored by direction of the effect, where red indicates higher abundance in patients and blue indicates depletion. HC, healthy controls; NAFL, non-alcoholic fatty liver; NASH, non-alcoholic steatohepatitis.

We then focused on a core set of the 38 most significant markers (FDR < 1 × 10^–3^) for further analysis (Figure 2A). Among these, *Enterococcus faecalis* was previously identified enriched in liver fibrosis patients (Aron-Wisnewsky et al., 2020), and the rest were new microbial biomarkers. Strains enriched in NAFLD such as *Megasphaera unclassified*, *Streptococcus* sp. *I G2*, *Clostridium spiroforme*, *Coriobacteriaceae bacterium phI*, *Turicibacter* sp. *HGF1*, *Veillonella* sp. *3 1 44*, *Streptococcus sanguinis*, and *Streptococcus anginosus* negatively correlated with species enriched in healthy controls, including *Weissella cibaria*, *Saccharomyces unclassified*, *Lactobacillus vaginalis*, *Streptococcus* sp. *GMD4S*, *Lactobacillus crispatus*, *Coprobacillus* sp. *8 2 54BFAA*, *Coprococcus* sp. *HPP0048*, and *Klebsiella unclassified* (Figure 3A). All of the classified NAFLD-enriched species are well-known pathogenic bacterium; for example, *C. spiroforme* toxin shows cytotoxicity and causes enteric diseases in human (Uzal et al., 2018), *S. sanguinis* is notorious for a cause of infective endocarditis (Zhu et al., 2018), and *S. anginosus* plays a pathogenic role in cystic fibrosis (Asam and Spellerberg, 2014). The majority of the control-enriched species are beneficial bacterium, for example, *W. cibaria*, as a kimchi lactic acid bacteria, has the ability to prevent cancer (Kwak et al., 2014) and *L. crispatus* is known as a biomarker of the healthy vaginal tract (Lepargneur, 2016; Veščičík et al., 2020). Another cluster of strains enriched in NAFLD including *Rothia dentocariosa*, *Bifidobacterium dentium*, *Actinomyces* sp. *ICM47*, *E. faecalis*, *Streptococcus* sp. *2 1 36FAA*, *Klebsiella oxytoca*, and *Streptococcus intermedius* negatively correlated with species enriched in healthy controls, including *Alistipes* sp. *JC136*, *Parabacteroides* sp. *ASF519*, *Streptococcus infantarius*, and *Dialister succinatiphilus* (Figure 3A). Most of the NAFLD-enriched species are pathogenic; for example, *R. dentocariosa* is a well-known causative agent of dental plaques and periodontal disease (Yang et al., 2007); *B. dentium* is the only single species of Bifidobacterium recognized as pathogenic (Meile et al., 2008); and *K. oxytoca* and *S. intermedius* were reported to be enriched in patients with angiocardiopathy (Jie et al., 2017). To determine whether specific microorganisms were correlated with NAFLD progression, we assessed the abundance of prokaryotes across the four stages of NAFLD by partial Spearman’s rank-based correlation (pSRC). At the species level, *C. spiroforme*, *Turicibacter* sp., *E. dolichum*, *L. salivarius*, *M. funiformis*, *S. anginosus*, *S. sanguinis*, *Streptococcus* sp., *V. dispar*, and *Veillonella* sp. were significantly correlated with NAFLD progression (pSRC, FDR < 0.05, Figure 3B), whereas *A. senegalensis*, *Coprobacillus* sp., *Coprococcus* sp., *L. crispatus*, *L. vaginalis*, and *W. cibaria* were significantly anti-correlated with NAFLD progression (pSRC, FDR < 0.05, Figure 3C).

In addition to the species associated to general microbiome dysbiosis for NAFLD, we also look for species associated with progressive subtype (NASH, fibrosis, and cirrhosis) that can lead to serious consequences, such as hepatocellular carcinoma and liver-related death. Ten species were enriched in patients with progressive NAFLD, including *Clostridium perfringens*, *Bacteroides coprophilus*, *Flavonifractor unclassified*, *Turicibacter sanguinis*, *Actinomyces graevenitzii*, *Anaerostipes unclassified*, *Coprobacillus* sp. *3 3 56FAA*, *Parabacteroides* sp. *D25*, *Eubacterium cylindroides*, and *Paraprevotella xylaniphila*, while five species were deleted, namely, *Streptococcus tigurinus*, *Slackia piriformis*, *Granulicatella adiacens*, *Roseburia unclassified*, and *Anaerostipes hadrus* (Figure 3D). *C. perfringens*, a well-known pathogen with the ability to secrete an arsenal of more than 20 virulent toxins (Kiu and Hall, 2018), is increased in several food poisoning and non-foodborne diseases. In our study, it is significantly and most increased in patients with fibrosis and cirrhosis, implying that the increase in the level of toxins can enter systemic circulation to affect various organs, such as liver. The higher level of *G. adiacens* has been reported to be associated with lung cancer (Shirazi et al., 2019) and pancreatic cancer (Memba et al., 2017), but in our study, it is decreased in patients with advanced NAFLD. Other strains have been little studied so far, so the role of microbe community in the development or prevention of disease is still an ongoing area of research.

We also did univariate analysis of genus associated with NAFLD. The raw counts were log-cpm-SNM transformed to reduce batch effect as mentioned above (Supplementary Figures 1A,B). At a meta-analysis FDR of 0.05, we identified 6 microbial species to be differentially enriched and 11 microbial species to be differentially depleted in the NAFLD patients out of 81 species consistently detected across studies (Supplementary Figure 3C). The gut microbial community also had consistent alteration patterns across different disease stages. *Megasphaera* has been reported to be enriched in patients with liver fibrosis (Chen et al., 2011), and we also got the same results in our study. *Porphyromonas* (Zhu et al., 2013) and *Peptoniphilus* (Del Chierico et al., 2017) were reported to be increased in patients with NASH, which was not repeated in our study, mainly owing to the sequence method and the sample from children. *Phascolarctobacterium* (Aron-Wisnewsky et al., 2020) were reported to be deceased in patients with obesity, a common complication of NAFLD, and we also got the same results in the present study. At the genus level, *Coriobacteriaceae noname*, *Fusobacterium*, *Megasphaera*, and *Sutterellaceae unclassified* were significantly correlated with NAFLD progression (Supplementary Figure 3D), and *Leuconostoc*, *Saccharomyces*, and *Weissella* were significantly anti-correlated with NAFLD progression (Supplementary Figure 3E).

### Functional Metagenomic Signatures for Non-alcoholic Fatty Liver Disease

**FIGURE 4:**
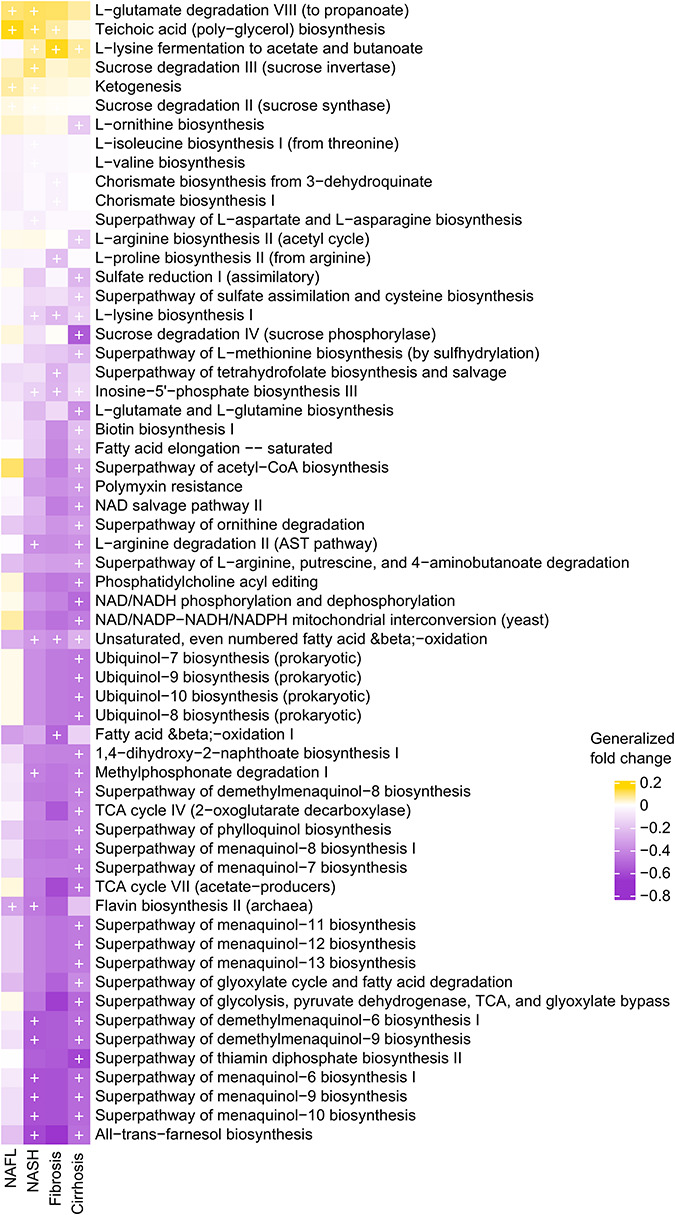
Microbial metabolic pathways altered in NAFLD. The significance of gut microbial species derived from blocked two-sided Wilcoxon tests (+ indicates statistical significance FDR < 0.05). In the generalized fold-change color scale, yellow represents microbial pathways that were increased in the NAFLD group compared with the healthy control group, while purple represents pathways that were decreased in the NAFLD group compared with the healthy control group. NAFL, non-alcoholic fatty liver; NASH, non-alcoholic steatohepatitis.

Functional potential of the microbiome was also significantly associated with NAFLD samples when compared to healthy controls. We found 11,941 of the 17,426 single gene families (FDR < 0.05) detected at least once to be enriched in NAFLD patients and 12,969 to be enriched in controls at meta-analysis FDR < 0.05. We further observed 179 out of 189 metagenomically reconstructed microbial functional pathways (FDR < 0.05) to be at least once control-enriched, and only 10 to be enriched in NAFLD patients at all stages of NAFLD. The disordered metabolic pathways showed an abnormal glycolipid metabolism, such as glycolysis, glyoxylate bypass, tricarboxylic acid (TCA) cycle, as well as fatty acid elongation, oxidation, and degradation, and these have been reported in several intestinal and metabolic disorders of multiple etiologies, such as colorectal cancer (Wirbel et al., 2019), obesity (Hoyles et al., 2018), as well as cardiovascular disease (Zhernakova et al., 2018). The NAFLD-enriched pathway is mainly associated with the production of harmful products, such as L-glutamate degradation VIII (to propanoate) and teichoic acid (poly-glycerol) biosynthesis (Figure 4). Propanoate generated from L-glutamate degradation is of hypotoxicity and inhibition of fatty acid synthesis (Hoke et al., 2016). The teichoic acid is a special component of the cell wall of gram-positive (G+) bacterium, helping bacteria stick to the surface of human cells, avoiding phagocytosis by leukocytes and resisting complement, which may be related to pathogenicity (Engholm et al., 2017). Most of the enriched species in patients gut microbiome are gram positive, such as *Streptococcus*, *Lactobacillus*, *Clostridium*, *Bifidobacterium*, and *Actinomyces*, which reinforce our finding. The significant alteration pathways also showed an association with NADH/NAD+ balance *via* glycolysis, TCA, and glyoxylate bypass, which have previously been associated with alcoholic hepatitis (Seronello et al., 2010) and metabolic dysfunction (Alves-Paiva et al., 2018; Figure 4). Butyrate is well known not only to be the preferred fuel for the colonic epithelial cells and the major regulator of cell proliferation and differentiation (Guilloteau et al., 2010), but also shown to exert important actions related to cellular homeostasis such as anti-inflammatory, antioxidant, and anti-carcinogenic functions (Hamer et al., 2009). Among pathways involved in butyrate production, the acetyl-CoA biosynthesis, L-glutamate biosynthesis, L-lysine biosynthesis, and L-histidine biosynthesis pathways were decreased in NAFLD patients (Figure 4), indicating the reduced protection effects of butyrate. The production of phosphatidylcholine, the main phospholipid in cellular membranes, from lysophosphatidylcholine acylation is a key component of the acyl-editing process, involving recycling of the fatty acids (Agarwal and Garg, 2010). Phosphatidylcholine provides the majority of the exogenous choline, the lack of which is linked to the accumulation of hepatic lipid and induced models of NAFLD in animals (Sherriff et al., 2016). The decreased phosphatidylcholine acyl editing pathway in progressive stages of NAFLD patients implied that the lack of protective effect of phosphatidylcholine and deficiency of choline might contribute to the liver injury (Figure 4). In addition, the pathway for synthesizing nutrients, such as menaquinol, flavin, ubiquinol, and phylloquinol, was observed to be reduced in patients (Figure 4). In addition, some species that were deleted in patients might affect Type II immune response. For example, *D. succinatiphilus*, known as succinate-utilizing bacteria, might regulate the phenotype and function of immune cells.

### Validation of Non-alcoholic Fatty Liver Disease Microbiome Signature

**FIGURE 5:**
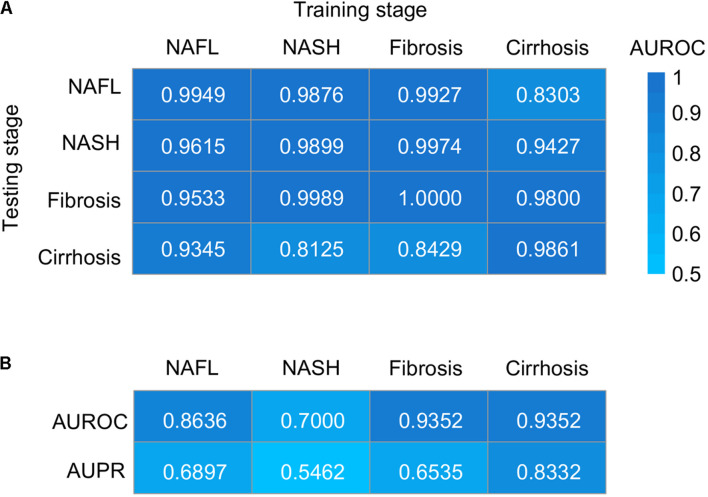
Taxonomic classification models generalize across stages by GBM. **(A)** Classification accuracy resulting from validation within each stage (along the diagonal) and stage-to-stage model transfer (external validations off-diagonal) as measured by AUROC. **(B)** Classification accuracy resulting from the models designed to distinguish patients with one stage of NAFLD from other stages. AUROC, area under the receiver operating characteristic curve; AUPR, area under the precision–recall curve; NAFL, non-alcoholic fatty liver; NASH, non-alcoholic steatohepatitis.

To evaluate the utility of the metagenomic gut microbiome signature for the detection of NAFLD, we tested its diagnostic accuracy between patients and healthy controls and cross four stages of NAFLD by training stochastic gradient-boosting machine (GBM) learning models. In patients–controls validation using species-level taxonomic normalized abundances, we observed performances ranging in area under the receiver operating characteristic curve (AUROC) score from 0.9861 to 1.0000 and in area under the precision-recall curve (AUPR) score from 0.8723 to 1.0000 (Figure 5A and Supplementary Figures 4A–D), and the performances were generally maintained in stage-to-stage transfer (AUROC ranged between 0.8125 and 0.9974) (Figure 5A). These results show that within-stage validation AUROCs and AUPR can be high for prediction of a certain stage of NAFLD with healthy controls, but they can also distinguish other stages from healthy controls, highlighting the homogeneous microbiome dysbiosis across stages. Given the clinical importance of our study, we further assessed whether it could discriminate one stage from the others. Encouragingly, we observed performances ranging in AUROC score from 0.7000 to 0.9352 and in AUPR score from 0.5462 to 0.8332 (Figure 5B and Supplementary Figures 4E–H), which performed well for discriminating between one stage and the others. Random forest model for verification was also used, and the classification performance was similar to GBM (Supplementary Figure 5). To evaluate the generalizability of our approach across datasets, we randomly sorted raw microbial counts into two batches, repeated all procedures on each independently, tested each independently trained model on the other half of the data, and found highly similar performance (Supplementary Figure 5). The slight reduction in sensitivities and specificities for split data may be partially due to small data sizes.

**FIGURE 6:**
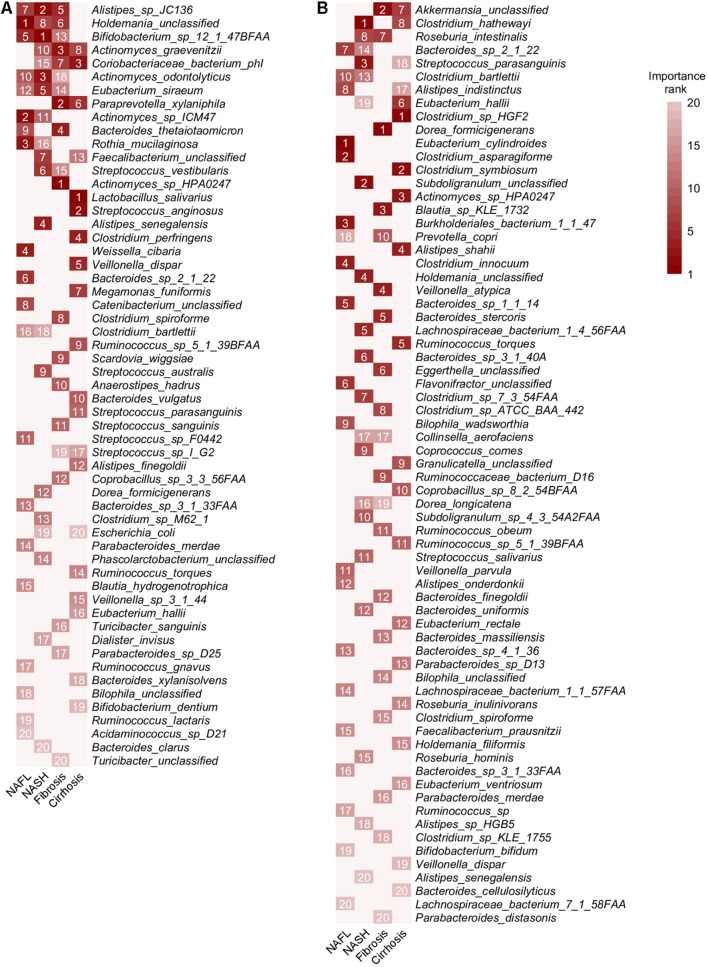
Ranking relevance of each species in the predictive models for each stage. **(A)** The importance of each species for the prediction performance in each dataset estimated using stochastic gradient-boosting machine (GBM) learning models. **(B)** The importance of each species for the prediction performance of the models designed to distinguish one stage of NAFLD from other stages. Only species appearing in the 20 top-ranking features in at least one dataset are reported. HC, healthy controls; NAFL, non-alcoholic fatty liver; NASH, non-alcoholic steatohepatitis.

Among the features used in the model validating patients and controls, *Alistipes* sp. *JC136* was the species with the highest average rank for the importance. As expected, other generally NAFLD-associated species including *Holdemania unclassified*, *Bifidobacterium* sp. *12 1 47BFAA*, *Coriobacteriaceae bacterium phI*, *A. odontolyticus*, *Eubacterium siraeum*, *Actinomyces* sp. *ICM47*, *Bacteroides thetaiotaomicron*, *R. mucilaginosa*, and *S. vestibularis* were also crucial to prediction accuracy (Figures 2, 6A). *A. graevenitzii* and *P. xylaniphila* were identified as species associated with advanced stages of NAFLD and also had good performance for validating patients and controls (Figures 3D, 6A). Among the features used in the model validating four stages of NAFLD, *E. cylindroides*, *Clostridium hathewayi*, *Dorea formicigenerans*, and *Clostridium* sp. *HGF2* had the highest rank for the importance, respectively (Figure 6B). Our previous study has shown that *C. hathewayi* was enriched in ankylosing spondylitis (AS) patients (Xu and Yin, 2019; Yin et al., 2020), but in this current study, we found that its abundance was significantly decreased in NASH patients with good discriminating performance.

## Discussion

Our study was performed across multiple datasets and populations, through a combined analysis of fecal NAFLD metagenomes from three publicly available datasets. Divergence of metagenomic approaches and study design, such as differences in sample collection and preservation, DNA extraction methodology and sequencing platform, all affect the composition of downstream sequence data. The effect of study-associated heterogeneity on microbiome composition was first quantified. The sequencing platform was the same in all three studies (Illumina HiSeq), while DNA extraction methods were different. Although all three studies stated that the sampling method was rapid freezing to −80°C, there were still technical differences due to human factors. So, the technical variation, such as sampling and DNA extraction for each of the downloaded dataset, was integrally considered as batch effects, which be dealt with at the beginning. Although these effects cannot be completely eliminated, they were greatly reduced (Figure 1B).

Researchers are often more likely to focus on the difference between disease and healthy controls, while the commonality between related diseases is often neglected. Here, we identified a core gut microbiome signature for general NAFLD microbiome dysbiosis instead of disease-stage-specific links (Figure 2). Although most of the classified NAFLD-enriched species have been shown to be pathogenic bacterium for human or animal models (Yang et al., 2007; Meile et al., 2008; Asam and Spellerberg, 2014; Uzal et al., 2018; Zhu et al., 2018), they were first to be shown as species associated with NAFLD. Among these, we demonstrate an interaction pattern of the most significant set of species (Figure 3A), and most of them were significantly correlated or anti-correlated with disease progression (Figures 3B,C). Boursier et al. (2016) had reported that *Ruminococcus* was significantly enriched in patients with both NASH or fibrosis, and in this current study, we also found a consistent result. Two *Ruminococcus* species (*Ruminococcus gnavus* and *Ruminococcus* sp. *JC304*) were identified as biomarkers for general NAFLD microbiome dysbiosis, and both were enriched in patients whatever stage (Figure 2). *Coprococcus* were reported to be decreased in NASH patients in three studies (Zhu et al., 2013; Wang et al., 2016; Hoyles et al., 2018). In our present study, three *Coprococcus* species (*Coprococcus catus*, *Coprococcus* sp. *ART55 1*, and *Coprococcus* sp. *HPP0048*) were found to be associated with general NAFLD microbiome dysbiosis, and all of them were commonly decreased in all stages of NAFLD patients (Figure 2). Furthermore, we described the species and metabolic pathways that specifically associated with progressive stages of NAFLD, implying that the increased toxic metabolites and decreased protection of butyrate and choline (Figures 3D, 4) together with diseased α-diversity (Figure 1C) contribute to NAFLD progression.

Broadly applicable, non-invasive methods for diagnosing the stage of NAFLD are currently not available. The identification of microbial biomarkers for NAFLD may enable the design of non-invasive diagnostic tools. We developed machine learning models able to distinguish patients with any stage of NAFLD from healthy controls with an average performance of 0.99 AUROC when validated on datasets excluded from the training of the model (Figure 5A). The models designed to distinguish patients with one stage of NAFLD from other stages also had an excellent performance with an average AUROC of 0.8585 (Figure 5B). The slight reduction of AUROC for the later model compared to the former indicate more homogeneity in microbiome across four stages of NAFLD than with healthy control, which was also verified by principal coordinate analysis (Figure 1D) and the consistent alteration across NAFLD stages (Figure 2). Therefore, microbiome-based NAFLD prediction models enable a very high diagnostic potential. The integrated data are slightly unbalanced between case and control, so we choose GBM, a decision tree-based integrated learning. Decision trees tend to do well with category disequilibrium data. It uses categorization rules based on class variables to create a classification tree so that samples of different categories can be forcibly separated. The training set of category unbalanced data has little influence on the training results of decision tree algorithms. Furthermore, if these diagnostic features can distinguish non-alcoholic fatty liver and alcoholic fatty liver or other similar diseases such as liver cirrhosis caused by other causes, it will be more significant. However, shotgun sequencing is expensive, and related researches are also very few. Therefore, it is hard to obtain related available data. As the price of sequencing falls and more data become available, this may become possible.

Although this study included the relatively small sizes of the experimental cohorts, analysis of patients with different stage of NAFLD presents a distinct opportunity for studying the general NAFLD-associated and stage-specific microbiome. By combining multiple cohorts of potentially low generalizability, it is possible to obtain better representation of the spectrum of NAFLD cases and controls. At present, researches about gut microbes are still very limited, and we still know little about the role of different strains in different situations. Even some known probiotics can be opportunistic pathogens. Therefore, this study combines data from three studies to identify potential candidate bacteria that contribute to disease development. These bacteria have been poorly studied and functional studies are needed to explore their role in disease. With appropriate methodology, artifactual findings due to batch effects present in any individual dataset can be avoided. In addition, the identification of pathogenic and beneficial microbial species might lead to novel therapies for severe forms of NAFLD. Taking account of limited accuracy of serum markers, the expense of MRE technologies and the invasiveness of liver biopsy, gut microbiome test is more convenient and feasible for disease screening. Our discovery of a gut microbiome-derived signature that accurately identifies the stage of NAFLD lays the foundations and points to the potential for non-invasive microbial diagnostic tests to supplement existing screening.

## Materials and Methods

### Study Inclusion and Data Acquisition

We used PubMed to search for studies that published fecal shotgun metagenomic data of human NAFLD patients and healthy CTRLs. Raw FASTQ files were downloaded for the three included studies from the European Nucleotide Archive (ENA) using the following ENA identifiers: ERP015847 for Hoyles et al. (2018), PRJNA373901 for Loomba et al. (2017), and ERP005860 for Qin et al. (2014).

### Sample Processing and Filtration

The stage of NAFLD was diagnosed according to liver biopsy. Biopsies were assessed for the following three parameters: Steatosis was graded 0–3, lobular inflammation was graded 0–3, and ballooning was graded 0–2. Fibrosis stage was classified into five stages from 0 to 4. NAFL patients have fat accumulation in the liver (steatosis) involving at least 5% of hepatocytes on routine stains without lobular inflammation, ballooning, and fibrosis. Presence of NASH was defined as a pattern that was consistent with steatohepatitis including presence of at least 5% steatosis, lobular inflammation, and ballooning with or without peri-sinusoidal fibrosis (fibrosis stage 1). Fibrosis stage consists of periportal fibrosis (fibrosis stage 2) and bridging fibrosis (fibrosis stage 3). Cirrhosis was defined as stage 4 fibrosis.

Participants were included in the study if they met the following criteria: (1) 18 years or older, (2) fat accumulation in the liver (steatosis) involving at least 5% of hepatocytes on routine stains, (3) no evidence of other acute or chronic liver disease, and (4) absence of regular or excessive use of alcohol. Patients were excluded from the study if they met any of the following criteria: (1) clinical or histological evidence of alcoholic liver disease; and (2) clinical or biochemical evidence of liver diseases other than NAFLD, including hepatitis B, hepatitis C, alpha-1 antitrypsin deficiency, hemochromatosis, Wilson’s disease, autoimmune hepatitis, polycystic liver diseases, cholestatic liver diseases, and vascular liver diseases. Patients in the three studies who met the above conditions and had a clear liver biopsy diagnosis were included in this study.

The liver imaging and liver biochemistry results of all healthy controls were in the normal range. Physical examination; routine examination of blood, urine, and stools; preoperative serological tests; liver function; renal function; electrolyte; liver ultrasound; electrocardiogram; and chest X-ray results were checked in the healthy controls to exclude any abnormal samples, such as clinical or biochemical evidence of liver diseases, chronic illnesses associated with hepatic steatosis, use of drugs known to cause hepatic steatosis, and presence of systemic infectious illnesses.

### Sequence Preprocessing and Taxonomic and Functional Profiling

Fecal metagenomic shotgun sequences were quality filtered using Trimmomatic (Bolger et al., 2014). Filtered reads were then aligned to the human genome and the PhiX genome for human and contaminant DNA removal using bowtie2 (Langmead and Salzberg, 2012). We used MetaPhlAn2 for quantitative profiling of the taxonomic composition of the microbial communities of all metagenomic samples, and HUMANn2 was chosen to profile pathway abundances using a ChocoPhlAn database and gene-family abundances using a UniRef90 database. Microbial profiles were first converted to count per million (cpm) to account for library size. Then, profiles were filtered to focus on a set of species that were detectable in all studies. Specifically, microbial species that did not exceed a cpm of 1 in at least three samples were excluded from further analysis. Functional profiles, such as gene family or metabolic pathway abundance profiles, were preprocessed as the species profiles.

### Normalization and Confounder Analysis

Cognizant of how technical variation and heterogeneous ethnicity between studies could confound our results, we made data normalization to remove batch effects before further analysis. In brief, we transformed our discrete taxonomical count data to approximately normally distributed, log-count per million (log-cpm) data, which models and removes the data’s heteroskedasticity; and then performed supervised normalization (SNM) on the data to remove significant batch effects while preserving biological effects (Mecham et al., 2010). Confounder analysis was performed by ANOVA to quantify the effect of confounders relative to that of disease state on single microbial species. Variance calculations were performed on ranks of microbiome abundance in non-Gaussian distribution.

### Statistical Analyses

Since microbiome data are characterized by non-Gaussian distributions with excessive dispersion, the non-parametric significance testing using blocked Wilcoxon rank-sum testing was implemented in the R “coin” package (Hothorn et al., 2006). Informed by the results of the preceding confounder analysis, BMI was blocked in meta-analysis. The mean difference in a set of predefined quantiles of the logarithmic case and distributions was calculated as a generalized fold change as this has been shown to achieve better resolution for sparse microbiome profiles than other methods (Feng et al., 2012). We used quantiles ranging from 0.1 to 0.9 in increments of 0.1. The non-parametric effect size was measured by Area Under Curve (AUC) obtained from the “pROC” package (Robin et al., 2011) in R. The co-occurrence of significantly different genera was analyzed using Spearman correlation. Partial Spearman’s rank-based correlation (pSRC) in the R “coin” package (Hothorn et al., 2006) was used to assess the associations between microbial profile and NAFLD stages with BMI as confounder. To adjust for multiple hypothesis testing, *p*-values were adjusted using the false discovery rate (FDR) method (Benjamini and Hochberg, 1995). Principal coordinate analyses based on species-level Bray–Curtis dissimilarity, together with α-diversity, were calculated using “vegan” in R (Dixon, 2003).

### Machine Learning Model

Stochastic GBM learning models were trained, automatically tuned, and tested using the GBM package (Friedman and Analysis, 2002) and Caret package (Kuhn, 2008) in R. Training and testing occurred on separate, randomly selected, stratified sampling splits of 70 and 30% of the data, respectively, and a fixed random number seed was used to ensure reproducibility of the model results and comparability among models. During model training, the data were first centered and scaled for each sample to have mean zero and unit standard deviation; fourfold cross-validation was used to create multiple subsets of the training data and to perform a basic grid search optimization of GBM parameters, including interaction depth (1, 2, or 3) and number of trees (50, 100, or 150), while maximizing AUROC of the final, fully trained model. Learning rate (shrinkage) was held constant at 0.1 and the number of minimum observations per node was fixed at 5. Final model performances, including ROC curves and PR curves, were generated by applying the final model to the unseen 30% holdout test set. ROC and PR curves as well as AUROC and AUPR values were calculated using the PRROC package (Grau et al., 2015). Variable importance scores of the resultant, non-zero model features were estimated using the GBM and Caret packages (Friedman and Analysis, 2002; Kuhn, 2008). Performance of classifiers within each stage was analyzed by fourfold cross-validation. Performance of classifiers across stages was analyzed by stage-to-stage transfer validation, that is, each stage was taken as training set, and the others were taken as testing sets. All species detected were included as features, and the importance of each feature was analyzed.

## Data Availability Statement

The datasets presented in this study can be found in online repositories. The names of the repository/repositories and accession number(s) can be found in the article/Supplementary Material.

## Author Contributions

TW and HX conceived and supervised the study. TW performed the taxonomic profiling, machine learning, statistical analyses, produced the figures, and wrote the manuscript with contributions from X-KG and HX. All authors discussed and approved the manuscript.

## Conflict of Interest

The authors declare that the research was conducted in the absence of any commercial or financial relationships that could be construed as a potential conflict of interest.

## Publisher’s Note

All claims expressed in this article are solely those of the authors and do not necessarily represent those of their affiliated organizations, or those of the publisher, the editors and the reviewers. Any product that may be evaluated in this article, or claim that may be made by its manufacturer, is not guaranteed or endorsed by the publisher.
