# Supplementary material for: Disentangling the Progression of Non-alcoholic Fatty Liver Disease in the Human Gut Microbiota
Source: Front Microbiol. 2021 Oct 13;12:728823. doi: 10.3389/fmicb.2021.728823 (PMC8548776; doi:10.3389/fmicb.2021.728823)
Supplement: Supplementary file 1 [file Data_Sheet_1.PDF]

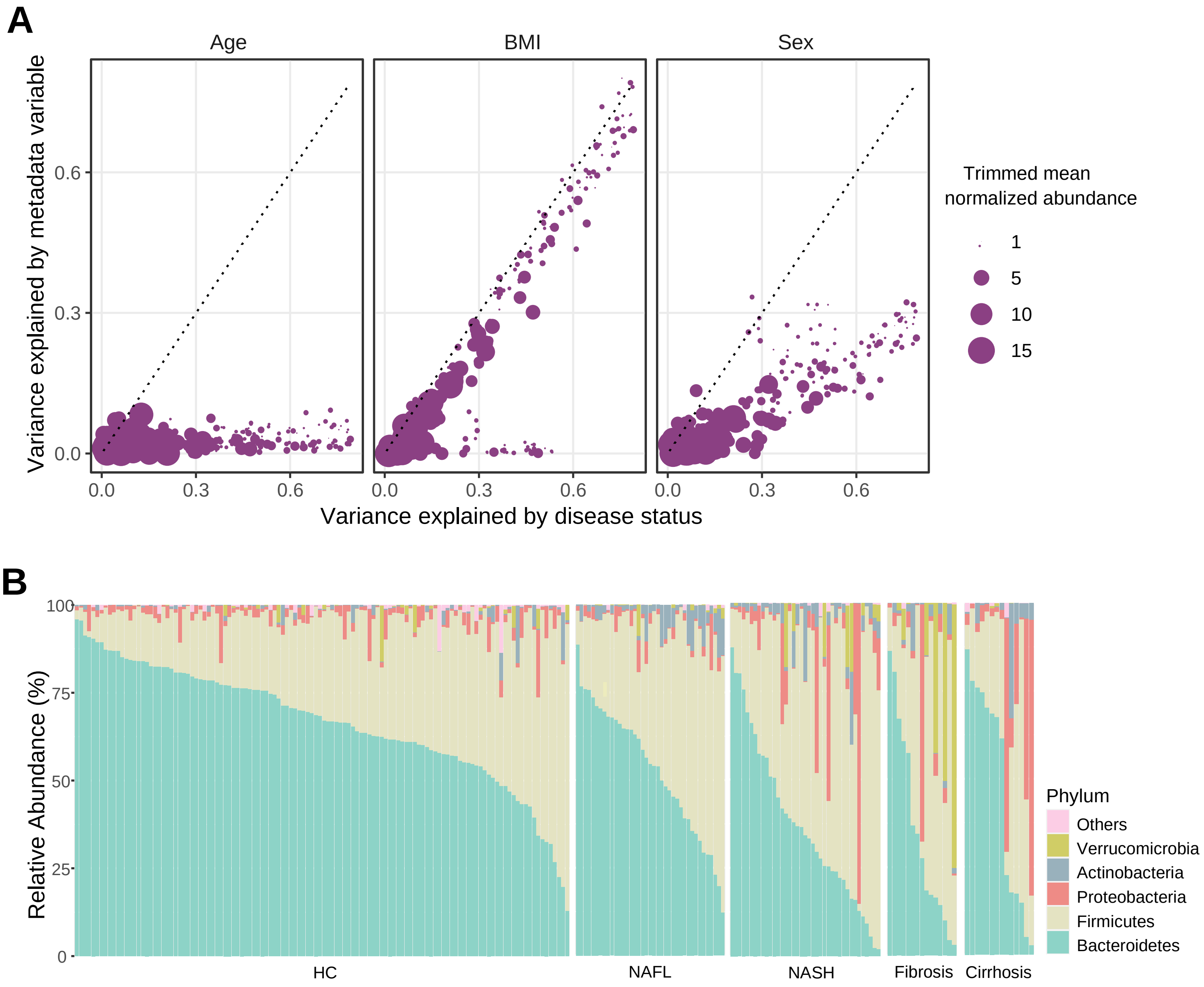

Supplementary Figure 1: Confounder analysis and microbiome composition. (A) Variance explained by disease status (case versus control) is plotted against variance explained by potential confounding factor (age, BMI and sex) for individual microbial species. Each genus is represented by a dot proportional in size to its normalized abundance. (B) Stacked bar plots depicting phylum-level differences in gut microbiome composition between the NAFLD stages and healthy controls. HC, healthy controls; NAFL, nonalcoholic fatty liver; NASH, nonalcoholic steatohepatitis.

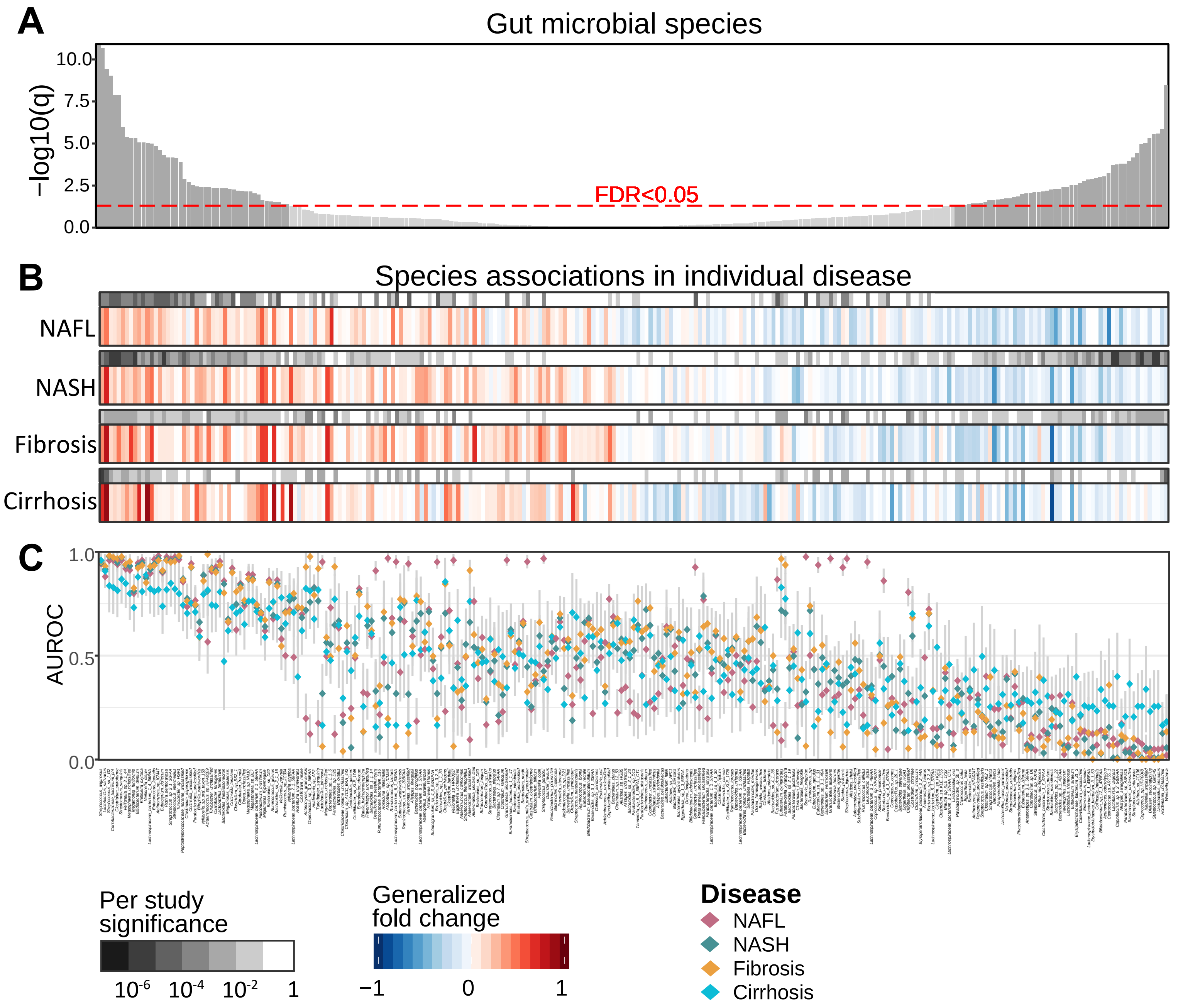

Supplementary Figure 2: Consistent alteration patterns across stages of species detected in all studies. (A) Meta-analysis significance of gut microbial species derived from blocked Wilcoxon tests is given by the bar height. Bars of a core of highly significant species (meta-analysis FDR = 0.05) were colored as dark gray. (B) Species-level significance, as calculated with a blocked two-sided Wilcoxon test (FDR P value), and the generalized fold change within individual stages are displayed as heatmaps in gray and in color, respectively. Species are ordered by meta-analysis significance and direction of change. (C) Association strength is quantified by the AUROC across individual stages (color-coded diamonds), and the 95% confidence intervals are indicated by the gray lines. Order-level taxonomic information is color-coded above the species names. HC, healthy controls; NAFL, nonalcoholic fatty liver; NASH, nonalcoholic steatohepatitis.

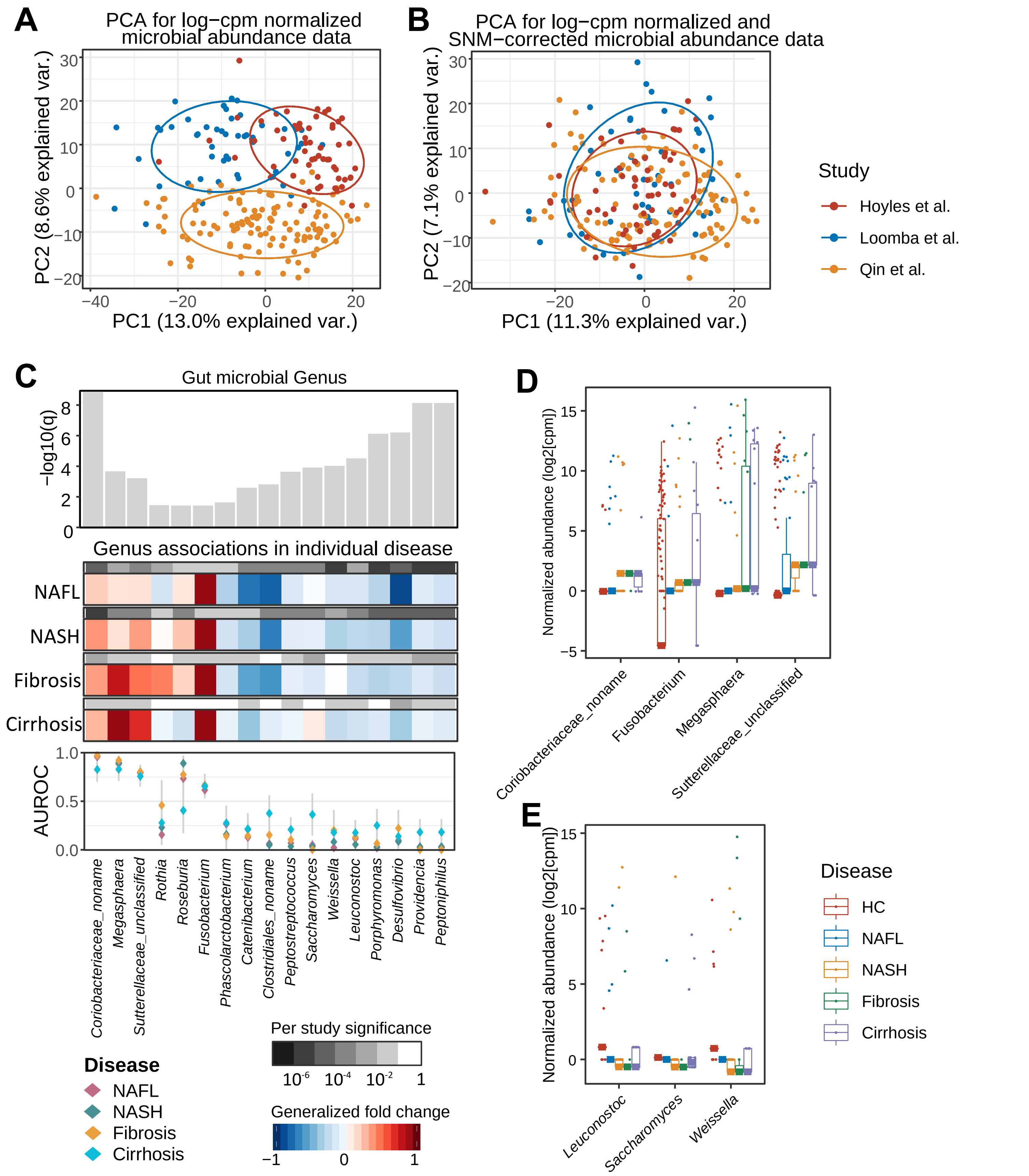

Supplementary Figure 3: Normalization and association of microbiome taxonomic profiles with NAFLD at genera level. (A) Principal components analysis (PCA) of log-cpm normalized data, with NAFLD microbiome samples colored by studies. (B) PCA of log-cpm -SNM data. (C) Meta-analysis significance of gut microbial genera derived from blocked Wilcoxon tests is given by the bar height (FDR = 0.05). Underneath, genera-level significance, as calculated with a blocked two-sided Wilcoxon test (FDR *P* value), and the generalized fold change within individual stages are displayed as heatmaps in gray and in color, respectively. Genera are ordered by meta-analysis significance and direction of change. Association strength is quantified by the AUROC across individual stages (color-coded diamonds), and the 95% confidence intervals are indicated by the gray lines. (D) Boxplots showing that genera was significantly correlated with NAFLD stages using partial Spearman's rank-based correlation (pSRC), adjusted for BMI (two-sided, FDR < 0.05;). (E) Boxplots showing that genera was significantly anti-correlated with NAFLD stages (pSRC, two-sided, FDR < 0.05). HC, healthy controls; NAFL, nonalcoholic fatty liver; NASH, nonalcoholic steatohepatitis.

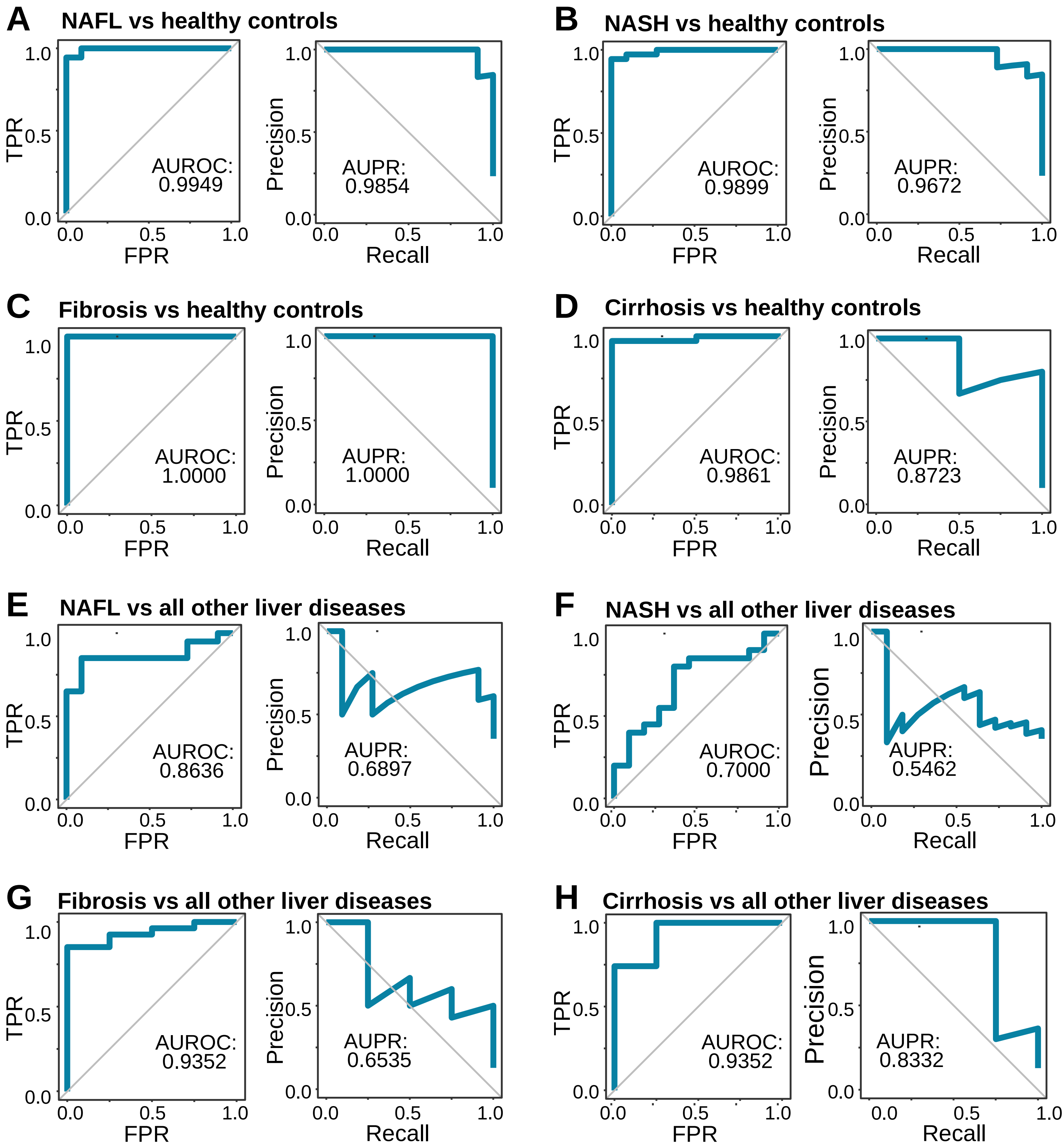

Supplementary Figure 4: Performances of machine learning models to discriminate within and between NAFLD stages. (A)-(D), Performances of models that distinguish a certain stage from healthy controls. (A) NAFL; (B) NASH; (C) fibrosis; (D) cirrhosis. (E)-(H), Performances of models that distinguish a certain stage from other stages. (E) NAFL; (F) NASH; (G) fibrosis; (H) cirrhosis. AUROC, area under the receiver operating characteristic curve; AUPR, area under the precision-recall curve; NAFL, nonalcoholic fatty liver; NASH, nonalcoholic steatohepatitis.

A

Training stage (columns) / Testing stage (rows) — AUROC

Testing stage \ Training stage	NAFL	NASH	Fibrosis	Cirrhosis
NAFL	0.9899	0.9966	0.9991	0.8699
NASH	0.9641	0.9975	0.9996	0.9585
Fibrosis	0.9533	1.0000	1.0000	0.9922
Cirrhosis	0.8583	0.8732	0.8988	0.9816

AUROC: 1, 0.9, 0.8, 0.7, 0.6, 0.5

B

	NAFL	NASH	Fibrosis	Cirrhosis
AUROC	0.8818	0.7477	0.9352	0.9444
AUPR	0.7597	0.7239	0.6535	0.8419

Supplementary Figure 5. Taxonomic classification models generalize across stages by random forest. (A) Classification accuracy resulting from validation within each stage (along the diagonal) and stage-to-stage model transfer (external validations off-diagonal) as measured by AUROC. (B) Classification accuracy resulting from the models designed to distinguish patients with one stage of NAFLD from other stages. AUROC, area under the receiver operating characteristic curve; AUPR, area under the precision-recall curve; NAFL, nonalcoholic fatty liver; NASH, nonalcoholic steatohepatitis.

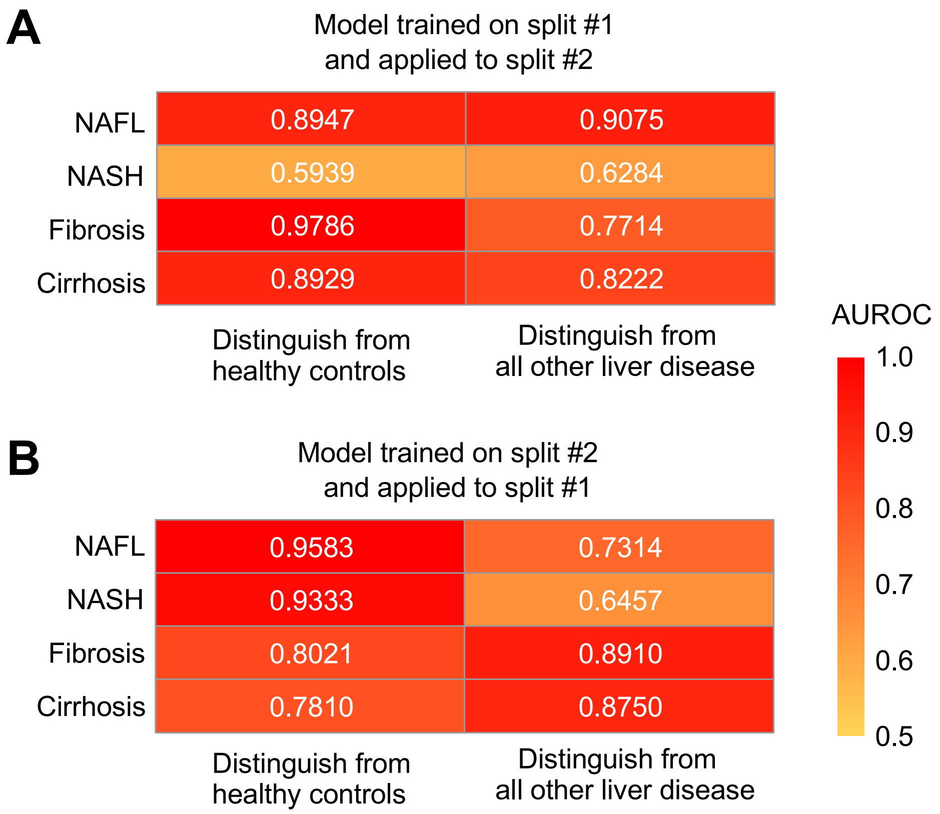

Supplementary Figure 6: Internal validation of ML model pipeline. Two independent halves of raw microbial count data were normalized and used for model training to predict one stage versus healthy controls (A) or all other stages (B); each model was then applied to the other half's normalized data. This heatmap compares the performances of these models compared to training and testing on 50–50% splits of the full data set.
